# Treatment of depression and anxiety with internet-based cognitive behavior therapy in patients with a recent myocardial infarction (U-CARE Heart): study protocol for a randomized controlled trial

**DOI:** 10.1186/s13063-015-0689-y

**Published:** 2015-04-11

**Authors:** Fredrika Norlund, Erik MG Olsson, Gunilla Burell, Emma Wallin, Claes Held

**Affiliations:** Department of Public Health and Caring Sciences, Uppsala University, Box 564, Uppsala, SE-751 22 Sweden; Department of Psychology, Uppsala University, Box 562 S-75122, Uppsala, Sweden; Department of Medical Sciences, Cardiology, Uppsala Clinical Research Center Uppsala University, Dag Hammarskjölds väg 14B, S-752 37 Uppsala, Sweden

**Keywords:** Myocardial infarction, CBT, iCBT, Anxiety, Depression, Internet, U-CARE

## Abstract

**Background:**

Major depression and depressive symptoms are common in patients with a recent myocardial infarction (MI), and depression is associated with adverse cardiovascular outcomes. Anxiety post-MI is less studied, but occurs commonly in patients with heart disease, and is also considered a risk factor for recurrence of cardiac events. Cognitive behavior therapy (CBT) is an established therapy for depression and anxiety disorders. To the best of our knowledge, there have not been any studies to determine if internet-based CBT (iCBT) can reduce the symptoms of depression and anxiety in patients with a recent MI. The main aim of the U-CARE Heart trial is to evaluate an iCBT intervention for patients with a recent MI.

**Methods/design:**

This is a randomized, controlled, prospective study with a multicenter design. A total of 500 participants will be randomized at a 1:1 ratio, around two months after an acute MI, to either iCBT or to a control group. Both groups will receive an optimal standard of care according to guidelines. The intervention consists of a self-help program delivered via the internet with individual online support from a psychologist. Treatment duration is 14 weeks. The primary outcome is change in patients’ self-rated anxiety and depression symptoms from baseline to end of treatment. An internal pilot study was conducted indicating sufficient levels of study acceptability and engagement in treatment.

**Discussion:**

The present study is designed to evaluate an iCBT intervention targeting symptoms of depression and anxiety in a post-MI population. If effective, iCBT has several advantages, and will potentially be implemented as an easily accessible treatment option added to modern standard of care.

**Trial registration:**

This trial was registered with Clinicaltrials.gov (identifier: NCT01504191) on 19 December 2011.

## Background

### Depression and anxiety is common in patients with myocardial infarction

Depression and depressive symptoms are common in patients with a recent myocardial infarction (MI). Major depression is reported to be prevalent in 20% of this patient population and, when including subsyndromal depressive symptoms, the prevalence is 31% [1]. Depression is also associated with adverse cardiovascular (CV) outcomes [2]. Furthermore, depression is a chronic, disabling condition associated with poor quality of life, functional limitation, less favorable self-care behaviors, and higher health care costs among patients with heart disease [3]. There are several possible mechanisms linking depression to cardiac events. Smoking and hypertension are known risk factors for heart disease that are more prevalent in depressed patients [4,5]. Depression is also associated with poor adherence to cardiac rehabilitation, which may lead to a less optimal recovery and worsened prognosis [6]. In addition, depression is an independent risk factor for future events. Possible mechanisms may be neuroendocrine dysfunction, disturbances in autonomic cardiac control, enhanced platelet activity, endothelial dysfunction, and inflammation [2].

Anxiety in patients with a recent MI and its association with prognosis has been less studied than depression, and the prevalence of patients diagnosed with anxiety is therefore uncertain. However, in one study, 38% of a post-MI group reported at least mild symptoms of anxiety, while only 18% in the same population reported at least mild symptoms of depression [7]. Importantly, in a large meta-analysis it was concluded that anxiety was not only common after MI, but also an independent risk factor for incident coronary heart disease and cardiac mortality [8]. Assumed mechanisms connecting anxiety and MI are more rapid progression of atherosclerosis, increased risk of ventricular arrhythmias, and unhealthier lifestyle factors, which are common among patients with anxiety and are known risk factors for heart disease [9]. Feelings of anxiety, sadness, and anger and/or hostility are highly correlated, and all are considered risk factors for first and recurrent cardiac events. It’s been suggested that at general disposition toward negative affectivity may be a more important risk factor than the expression of any specific negative affect [10].

### Psychological interventions for patients with a recent myocardial infarction

Psychological interventions may reduce the level of depression in cardiac patients according to a Cochrane review [11], and of the psychological interventions, cognitive behavioral therapy (CBT) has shown the most favorable results [12]. Despite the fact that anxiety is a risk factor for MI, very few treatment studies on anxiety in patients with a recent MI have been conducted [11]. To our knowledge, there is only one randomized controlled clinical trial (RCT), which showed no effect of the intervention [13]. There are several studies that have used group-based interventions focusing on a broader range of psychosocial risk factors with good results. These studies use CBT principles in a cognitive behavioral stress management program [14-16].

There is a high degree of spontaneous recovery of anxiety and depression in the first month after an MI [17]. By delaying the start of the treatment by two to three months after the acute event, one can exclude those who recover spontaneously from an initial depression or crisis and minimize the risk of treating people who will recover spontaneously. In the ENRICHD-study, therapy started two weeks after the MI, which led to substantial recovery in both the treatment group and the control group [18].

### Internet-based cognitive behavioral therapy

During the past decade, internet-delivered psychological interventions have become more common, and internet-based CBT (iCBT) has several advantages over traditional face-to-face CBT. First, the treatment can be delivered at any preferred time and place, and participants can work with the material at their own pace and review it as often as desired. Second, the level of therapist involvement can be adjusted and individualized; thus it may be possible to reduce the therapist time while maintaining efficacy [19]. Importantly, iCBT has been shown to be as effective as traditional CBT when it comes to anxiety and depression [19]. To achieve good compliance and results when delivering iCBT, individual support by psychologists is recommended [20]. It also seems beneficial to tailor the intervention towards specific problems in the target population instead of using generic treatments [21]. Although there are fewer studies available for iCBT, it seems to work well in populations with various somatic disorders [22]. To our knowledge, there are no RCTs available to determine the effects of iCBT in patients with a recent MI.

### Study objectives

The aim of the U-CARE Heart trial is to prospectively study the effects of iCBT in male and female patients with a recent MI with symptoms of depression and anxiety. Primary endpoints are change in self-rated depression and anxiety. Secondary endpoints are quality of life, stress behaviors, fatigue, sleep pattern, posttraumatic stress, and posttraumatic growth. Furthermore, we will evaluate health economy aspects and the cost-effectiveness of the intervention, and study its effect on later major adverse cardiac events.

## Methods/design

The present study is part of the Uppsala University Psychosocial Care Program (U-CARE), which aims at developing psychosocial care for somatic disease via the internet [23]. An internet platform (the U-CARE portal, Uppsala University, Uppsala, Sweden) has been developed within the U-CARE program. The U-CARE portal is used for data collection and interventions in all projects undertaken within the framework of the program.

### Study design and participants

**Figure 1:**
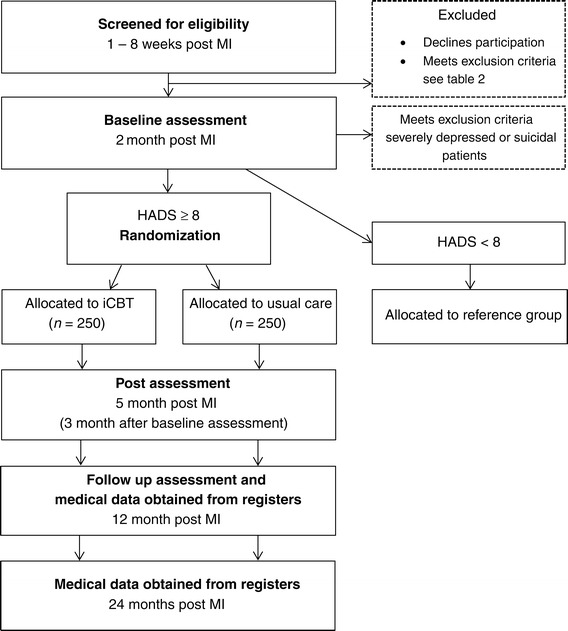
**U-CARE Heart overall study design.** HADS, Hospital Anxiety and Depression Scale; iCBT, internet-based cognitive behavioral therapy; MI, myocardial infarction.

U-CARE Heart is a prospective RCT. Figure 1 provides an outline of the trial design. A total of 500 participants will be recruited in a multicenter design, involving cardiology clinics in Sweden, in both rural and urban areas. Currently, 15 hospitals are engaged in the study. Inclusion and exclusion criteria are:

Inclusion criteria

- Age <75-years-old with a recent acute MI (<3 months)
- Depression and/or anxiety score of >7 on one or both of the Hospital Anxiety and Depression Scale (HADS) subscales [24].

Exclusion criteria

- Scheduled for a coronary artery bypass surgery
- Unable or unwilling to use a computer or internet, including email
- Unable or unwilling to use a mobile phone
- Difficulties in reading or understanding Swedish
- Life expectancy of less than a year
- Anticipated poor compliance
- Highly depressed or suicidal patients (Montgomery-Åsberg Depression Rating Scale Self Rating (MADRS-S) score >34 or MADRS-S item 9 > 3) [25]
- Participation in an ongoing trial with a behavioral intervention.

Participants scoring below eight on the HADS; will be allocated to a reference group which will be used to answer correlational research questions that are beyond the scope of this paper describing the RCT. Participants, both in the intervention group and in the control group, may seek other help such as psychopharmacology or counselling for their emotional problems if they wish to. Information on other treatment will be collected and controlled for in the statistical analyses. The first 20 randomized patients were included in an internal pilot study reported below. At that time of the pilot study, 12 hospitals were engaged in recruitment of participants.

### Statistical power, sample size and statistical principles

We expect an effect size of the treatment on symptoms of depression and anxiety of at least Cohen’s *d* = 0.30 (a small effect size). This is based on the results from the ENRICHD study [18]. With α = 0.05 and a power of 90%, a sample of 468 participants is needed, of which 234 will receive the intervention. Main results will be analyzed according to the intention-to-treat principle and thus include all participants. When analyzing secondary outcomes, Holm-Bonferroni correction will compensate for multiple comparisons. The study is underpowered to detect a potential effect on major adverse cardiac events and these analyses will only be exploratory.

### Randomization

Participants will be randomized (1:1) to either iCBT or standard of care. Randomization will be stratified by clinical center to obtain approximately equal sample sizes in both groups from every center.

### The internet-based cognitive behavioral therapy intervention

The iCBT intervention is inspired by face-to face CBT for psychological problems and earlier iCBT-manuals. CBT is a short-term therapy that aims at challenging negative patterns of cognitions and behaviors leading to negative feelings. It usually contains self-registrations and introduction of new, more functional, cognitions or behaviors. The iCBT intervention starts with a general introduction to the CBT model. The participants are asked to describe their present concerns and goals with their participation in the treatment. After completing the introduction, the participants will, with help from their therapist, choose the problem area(s) they want to work with. This self-tailored design is suggested to give the participants more control while maintaining treatment quality [26]. There are 10 treatment modules to choose from in the portal Each participant chooses two or three of the modules below after finishing the mandatory introduction to cognitive behavioral therapy:

- Worry management
- Fear and avoidance after a myocardial infarction
- Behavioral activation
- Problem solving
- Communication training
- Relaxation
- Cognitive restructuring
- Coping with insomnia
- Values in life
- Relapse prevention.

Each module contains two to four steps, and the participants are suggested to work with one step each week, for a total of 14 weeks. The treatment program includes texts, presentations, assignments, and self-monitoring, and offers a weekly feedback from a psychologist over the internet. The content of the modules were designed specifically for this study.

In addition, the participant can communicate with their psychologist through the portal at any time and ask for support or clarifications. Participants who do not log in or follow the treatment plan are reminded via automatic emails, text messages, telephone calls, or postal mail at their choice and are encouraged to continue.

The U-CARE portal also contains a discussion forum where participants have the possibility to communicate with each other, as well as videos with patients who have had an MI and who have struggled with negative feelings afterwards.

### Standard of care

All patients in the study are treated medically according to international guidelines by the local health care system regardless of treatment allocation. Standard of care includes secondary preventive interventions and cardiac rehabilitation activities according to local routines. This includes education about the disease and of risk factors, encouragement for lifestyle behavioral changes (for example, smoking cessation, diet modification, and increased physical activity), and psychosocial support. Psychotropic medication may be prescribed if considered necessary.

### Procedure

The patients are identified at the local hospitals one to eight weeks after their MI. The patients are contacted by project personnel 8 to 12 weeks after their MI and are sent an informed consent form for signing. After returning the signed consent form they are provided with login details. Each participant receives a username and password that they can change at their own choice. At each session they will also receive a text message with a five-digit numerical password to enter, in order to enhance security. Patients with signs of severe depression or reporting suicidal ideation when answering the baseline assessments will be excluded from the study and contacted via telephone by a project psychologist and, if needed, referred to adequate treatment. Likewise, if there is reason to believe that they have developed severe depression or have become suicidal during the study, patients will be contacted by the psychologist for assessment and possibly referred to other treatment. Reminders via email and text messages will be sent automatically from the platform if the questionnaires are not completed within seven days, and by telephone if they still have not been completed after another seven days.

The status of depression and/or anxiety for those in treatment will be monitored by the therapist via communication within the portal. If there are any indications of suicidal ideations or worsening of symptoms the therapist will contact the participant by telephone to evaluate their status, give advice on how to obtain other treatment or encourage them to carry on with their iCBT program.

### Data collection

#### Self-rating scales

All self-reported data will be collected via the U-CARE portal at three time points: (a) at baseline (two months after the MI), (b) immediately after program completion (14 weeks after baseline), and (c) during the follow-up period (12 months after the MI; Figure 1). Well-known and validated questionnaires with good psychometric properties reflecting various aspects on psychological wellbeing will be used.

##### Depression and anxiety

The main outcome measure assessed is self-reported depression and anxiety using the HADS [24], which comprises one subscale for anxiety symptoms and one for depressive symptoms. Values above seven points on any of the subscales indicate at least mild anxiety or depression. HADS has been criticized for measuring general distress rather than two separate symptom clusters [27]; however, anxiety and depressions are usually correlated even when other questionnaires are used [28]. The depression subscale of HADS has been shown to have prognostic value for morbidity and mortality in patients with acute coronary syndrome, even though somatic-affective symptom-items are lacking [29]. Depressive symptoms are also assessed by the Montgomery-Åsberg Depression Rating Scale-Self-rating (MADRS-S) [25]. The MADRS-S is also used to screen for severe depression and/or risk of suicide. To assess another aspect of depression, passivity and avoidance, the instrument Behavioral Activation for Depression Scale-Short Form is used [30]. Cardiac anxiety is assessed by the Cardiac Anxiety Questionnaire (CAQ) [31]. CAQ measures fear of, avoidance of, and focus on cardiac-related stimuli and sensations.

##### Other self-rating scales

Quality of life will be assessed by the EQ-5D [32] and the Cantril Ladder of Life scale [33]. Time urgency and hostility will be assessed by the Everyday Life Stress Scale [34], and vital exhaustion will be assessed using the Maastricht Questionnaire [35].Sleeping problems will be assessed using the Insomnia Severity Index [36], and posttraumatic stress will be assessed with Posttraumatic Stress Disorder Checklist- Civilian Version [37]. Posttraumatic growth, which refers to positive psychological change experienced as a result of struggle with highly challenging life circumstances, will be assessed with Posttraumatic Growth Inventory*-*Short Form [38].

#### Medical and demographic data

Data on CV morbidity and mortality will be collected based on information from national registers such as the Patient Administration Register and the Cause of Death Register. Clinical data will be obtained by the national SWEDEHEART registries, which is a framework for several quality assurance registers in Swedish CV health care. Events that will be accounted for are: overall and cardiac-related mortality, including cause of death; incidence of MI, revascularization and/or stroke; number of hospitalizations; and number of days in hospital. These data will be collected at 12 and 24 months post-MI for all participants in the study. Demographic data will be obtained via project-specific questions.

### Pilot study

#### Methods

An internal pilot study has been performed using the same procedures and design as in the main study, and an evaluation was made after randomization of the first 20 participants. At the time, 12 hospitals were engaged in the recruitment of participants. The objective of the pilot study was to evaluate the study acceptability and participant activity using two criteria; (1) at least 50% of eligible participants not meeting any exclusion criteria should accept participation in the study, and (2) at least 50% of the participants randomized to treatment should have submitted at least one homework assignment within three weeks. If these criteria were met, the pilot study would seamlessly proceed to the main study whilst retaining the pilot participants, in line with the methodology of internal pilot studies [39].

#### Results

**Figure 2:**
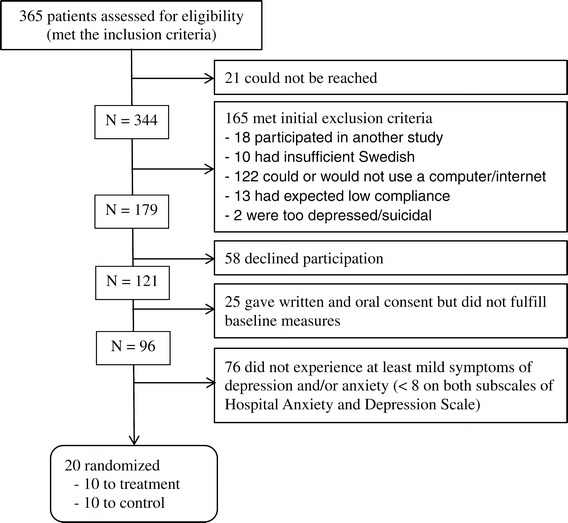
**Pilot study flow chart.**

The criteria for the pilot were met. Criterion one: 68% of the eligible patients not meeting any exclusion criteria accepted participation. The most common exclusion criterion, matching 35% of the eligible patients, was being unable or unwilling to use a computer or the internet. Of the consenting participants that completed the baseline questionnaires, 21% had HADS scores above seven and were randomized (see Figure 2). Criterion two: five participants (50%) submitted at least one homework assignment within three weeks from randomization. Another two participants submitted homework assignments eventually. Three participants were not active at all.

### Ethical considerations

The study has been approved by the Regional Ethical Review Board in Uppsala (approval number: 2011/217). Written informed consent will be obtained from the participants before inclusion. Extensive measures have been taken to minimize the risk of infringing activities, and to ensure that study participants’ personal data cannot be linked to patient-reported data by unauthorized persons. Study participants may at any time terminate participation without providing any explanation. An important ethical concern is to not include participants in the study who need other specialized medical care, such as those with severe depression; the baseline measurement is designed to minimize that risk. All study participants will also have an established contact with physicians in routine health care. If therapists receive information about unexpected coronary symptoms they are to inform the patient’s cardiac clinic after first consulting the study cardiologist.

## Discussion

The main result of the internal pilot study was that the two pre-specified criteria were met and the participants in the pilot study were included in the main ongoing study. Even though the criteria were met the data here presented indicate challenges both in recruitment and participant engagement. To reach the intended sample size when taking into account the relatively high rate of patients matching the exclusion criteria more hospitals will need to be engaged. Moreover, the power calculation we have made is conservative, using a high power and low effect size. A moderate effect size and acceptance of 80% statistical power will decrease the sample size needed substantially. We expected more participant engagement than what was evident from the pilot study. After the pilot study we made minor changes to our communication protocols at recruitment and when reminding patients. Minor changes were also made in the structure of the introduction module. These changes were intended to improve information dissemination and patient engagement. It would, however, be premature to draw conclusions about participant engagement from only 10 participants. Participant engagement is a recognized concern in iCBT [40] and patient motivation is considered the most important factor for engagement [41]. In the majority of iCBT studies participants have been recruited via media campaigns where they actively have sought treatment, whereas in this study patients were asked if they were interested by the hospital staff. The lack of externally valid studies is a major limitation, and therefore this study will clarify important questions of the feasibility of use of iCBT for patients with a recent MI in a clinical setting.

The present study is the first RCT using iCBT in patients with a recent MI with symptoms of depression and anxiety. If this program is successful, it will have important clinical implications and open up a possibility to satisfy an unmet need in these patients. Internet-based treatment has the potential for large-scale implementation at a reasonable cost. To increase the generalizability and opportunities for implementation nationwide, the study will be carried out in several counties and clinics across Sweden.

## Trial status

The inclusion of patients started in September 2013 and will continue until December 2016.

## Abbreviations

CAQ: Cardiac Anxiety Questionnaire
CBT: Cognitive behavioral therapy
CV: Cardiovascular
HADS: Hospital Anxiety and Depression Scale
iCBT: internet-based CBT
MADRS-S: Montgomery-Åsberg Depression Rating Scale-Self-rating
MI: Myocardial infarction
RCT: Randomized controlled clinical trial
U-CARE: Uppsala University Psychosocial Care Program
